# Prevalence and outcomes of atrial fibrillation in older people living in care homes in Wales: a routine data linkage study 2003–2018

**DOI:** 10.1093/ageing/afac252

**Published:** 2022-12-05

**Authors:** Leona A Ritchie, Stephanie L Harrison, Peter E Penson, Ashley Akbari, Fatemeh Torabi, Joe Hollinghurst, Daniel Harris, Oluwakayode B Oke, Asangaedem Akpan, Julian P Halcox, Sarah E Rodgers, Gregory Y H Lip, Deirdre A Lane

**Affiliations:** Liverpool Centre for Cardiovascular Science, University of Liverpool, Liverpool L7 8TX, UK; Department of Cardiovascular and Metabolic Medicine, Institute of Life Course and Medical Sciences, University of Liverpool, Liverpool L7 8TX, UK; Liverpool Centre for Cardiovascular Science, University of Liverpool, Liverpool L7 8TX, UK; Department of Cardiovascular and Metabolic Medicine, Institute of Life Course and Medical Sciences, University of Liverpool, Liverpool L7 8TX, UK; Liverpool Centre for Cardiovascular Science, University of Liverpool, Liverpool L7 8TX, UK; Department of Cardiovascular and Metabolic Medicine, Institute of Life Course and Medical Sciences, University of Liverpool, Liverpool L7 8TX, UK; School of Pharmacy and Biomolecular Sciences, Liverpool John Moores University, Liverpool L3 3AF, UK; Population Data Science, Health Data Research UK, Swansea University Medical School, Swansea University, Swansea, Wales SA2 8PP, UK; Population Data Science, Administrative Data Research Wales, Swansea University Medical School, Swansea University, Swansea, Wales SA2 8PP, UK; Population Data Science, Administrative Data Research Wales, Swansea University Medical School, Swansea University, Swansea, Wales SA2 8PP, UK; Population Data Science, Administrative Data Research Wales, Swansea University Medical School, Swansea University, Swansea, Wales SA2 8PP, UK; Population Data Science, Administrative Data Research Wales, Swansea University Medical School, Swansea University, Swansea, Wales SA2 8PP, UK; Liverpool Centre for Cardiovascular Science, University of Liverpool, Liverpool L7 8TX, UK; Department of Cardiovascular and Metabolic Medicine, Institute of Life Course and Medical Sciences, University of Liverpool, Liverpool L7 8TX, UK; Musculoskeletal and Ageing Science, Institute of Life Course and Medical Sciences, University of Liverpool, Liverpool L7 8TX, UK; Liverpool University Hospitals NHS Foundation Trust, Liverpool L9 7AL, UK; Population Data Science, Administrative Data Research Wales, Swansea University Medical School, Swansea University, Swansea, Wales SA2 8PP, UK; Department of Public Health, Policy and Systems, Institute of Population Health, University of Liverpool, Liverpool L69 3GF, UK; Liverpool Centre for Cardiovascular Science, University of Liverpool, Liverpool L7 8TX, UK; Department of Cardiovascular and Metabolic Medicine, Institute of Life Course and Medical Sciences, University of Liverpool, Liverpool L7 8TX, UK; Aalborg Thrombosis Research Unit, Department of Clinical Medicine, Aalborg University, Aalborg DK-9220, Denmark; Liverpool Heart and Chest Hospital, Liverpool L14 3PE, UK; Liverpool Centre for Cardiovascular Science, University of Liverpool, Liverpool L7 8TX, UK; Department of Cardiovascular and Metabolic Medicine, Institute of Life Course and Medical Sciences, University of Liverpool, Liverpool L7 8TX, UK; Aalborg Thrombosis Research Unit, Department of Clinical Medicine, Aalborg University, Aalborg DK-9220, Denmark; Liverpool Heart and Chest Hospital, Liverpool L14 3PE, UK

**Keywords:** Atrial fibrillation, stroke, care homes, prevalence, health outcomes, older people

## Abstract

**Objective:**

To determine atrial fibrillation (AF) prevalence and temporal trends, and examine associations between AF and risk of adverse health outcomes in older care home residents.

**Methods:**

Retrospective cohort study using anonymised linked data from the Secure Anonymised Information Linkage Databank on CARE home residents in Wales with AF (SAIL CARE-AF) between 2003 and 2018. Fine-Gray competing risk models were used to estimate the risk of health outcomes with mortality as a competing risk. Cox regression analyses were used to estimate the risk of mortality.

**Results:**

There were 86,602 older care home residents (median age 86.0 years [interquartile range 80.8–90.6]) who entered a care home between 2003 and 2018. When the pre-care home entry data extraction was standardised*,* the overall prevalence of AF was 17.4% (95% confidence interval 17.1–17.8) between 2010 and 2018. There was no significant change in the age- and sex-standardised prevalence of AF from 16.8% (15.9–17.9) in 2010 to 17.0% (16.1–18.0) in 2018. Residents with AF had a significantly higher risk of cardiovascular mortality (adjusted hazard ratio [HR] 1.27 [1.17–1.37], *P* < 0.001), all-cause mortality (adjusted HR 1.14 [1.11–1.17], *P* < 0.001), ischaemic stroke (adjusted sub-distribution HR 1.55 [1.36–1.76], *P* < 0.001) and cardiovascular hospitalisation (adjusted sub-distribution HR 1.28 [1.22–1.34], *P* < 0.001).

**Conclusions:**

Older care home residents with AF have an increased risk of adverse health outcomes, even when higher mortality rates and other confounders are accounted for. This re-iterates the need for appropriate oral anticoagulant prescription and optimal management of cardiovascular co-morbidities, irrespective of frailty status and predicted life expectancy.

## Key Points

- The prevalence of atrial fibrillation remained stable in older care home residents from 2010 to 2018.
- Residents with atrial fibrillation had a significantly higher risk of all-cause and cardiovascular mortality.
- The risk of stroke and cardiovascular hospitalisation was also significantly higher, even when mortality was adjusted for as a competing risk.
- Optimal management of atrial fibrillation in older care home residents is critical to improve health outcomes.

## Introduction

Older care home residents are a high-risk group of people with atrial fibrillation (AF) who are underrepresented in clinical trials. Improved understanding of AF epidemiology and management in this population is paramount. The health and social care burden of AF and AF-associated adverse health outcomes presents as a major public health issue, but the burden of AF in the care home setting is unclear. Estimates of AF prevalence in care homes have been reported to range from 7 to 38%, likely as a result of diversity and variability across residents and facilities [1]. Furthermore, in a recent systematic review on AF in care homes, only one study was identified that examined health outcomes in residents with AF compared to those without AF [1, 2]. This study only reported data on ischaemic stroke events, and did not report the confounders it adjusted for [2]. It is important to examine associations between AF and adverse outcomes in older care home residents because they have a high prevalence of multimorbidity, frailty, polypharmacy and dementia, which may impact the overall risk and whether oral anticoagulants (OACs) are prescribed. Treatment decisions are complex and require individualised assessment of the net-clinical benefit, especially in the context of guarded prognoses. Furthermore, older care home residents in the UK experience higher mortality rates compared with older community-dwelling residents [3], but different health outcomes of care home residents with AF have not been investigated by adjusting for the competing risk of mortality. Routinely linked data are valuable to address the paucity of available data on this vulnerable, high-risk group of people who are underrepresented in research [4, 5].

This study aims to use anonymised, individual-level population-scale routinely linked data for older people aged ≥65 years living in care homes in Wales to determine the prevalence of AF and temporal trends by year of care home entry, and associations between AF and adverse health outcomes including stroke, transient ischaemic attack (TIA), major bleeding, myocardial infarction (MI), cardiovascular hospitalisation and mortality.

## Methods

### Study design

The Secure Anonymised Information Linkage (SAIL) Databank project on CARE home residents with AF (SAIL CARE-AF) was a retrospective cohort study of anonymised, individual-level data on care home residents in Wales, provisioned from 1 January 2003 to 31 December 2018 and conducted following the REporting of studies Conducted using Observational Routinely-collected health Data (RECORD) 2015 guidelines [6] (Appendix 1).

### Data sources

This study utilised data available from the SAIL Databank [7–9]. The SAIL Databank contains multiple linked anonymised, population-scale routinely collected electronic health record and administrative data sources. This includes the Welsh Demographic Service Dataset [10], the Welsh Longitudinal General Practice (WLGP) [11] and the Patient Episode Database for Wales (PEDW) [12]. International Classification of Diseases version 10 (ICD-10) and Read version 2 codes were used to extract data from the PEDW and WLGP, respectively (Appendices 2 and 3). The WLGP data used by this study contain primary care data with ~80% coverage of patients and general practices in Wales. The PEDW secondary care dataset has 100% coverage of patients and services.

### Participants

The care home dataset (CARE) within the SAIL Databank relies on care home information available from the Care Inspectorate Wales (CIW) registry. In Wales, care homes must be registered with CIW who are responsible for inspection and quality improvement. Care homes include residential and nursing homes, which provide different levels of assistance to residents. Nursing homes provide a higher level of care and greater assistance to residents with the support of qualified nurses. The CIW registry 2017/18 was used in this study [13]. This was linked to anonymised address data for individual participants [14–16]. Data were extracted for people aged ≥65 years who had moved to a care home between 1 January 2003 and 31 December 2018. All participants had a minimum of 12 months of data coverage within the WLGP prior to moving to a care home. Complete linked data were available from 1 January 2000 for the entire cohort. The cohort was restricted to the first care home entry date to prevent participants being accounted for more than once if they moved in and out of different care homes. Participants were stratified by prior diagnoses of AF (any sub-type or atrial flutter) within the PEDW or WLGP (Appendices 2 and 3). The date of AF diagnosis was extracted, in addition to the date of the most recent record of AF contained within the WLGP or PEDW.

### Co-variates

Demographic covariates included week of birth, sex and the Welsh Index of Multiple Deprivation (WIMD) data (Version 2011) from the Welsh Government. The WIMD has five quintiles representing relative deprivation for areas in Wales, the most deprived (quintile 1) and the least deprived (quintile 5) [17]. Frailty assessment relied on the use of the Electronic Frailty Index (eFI) [18], calculated using 36 variables (referred to as ‘deficits’) [19]. Participants were categorised on care home entry according to frailty status: no frailty (eFI 0–0.12); mild (eFI >0.12–0.24); moderate (eFI >0.24–0.36) or severe frailty (eFI >0.36). Stroke and bleeding risk were also assessed on care entry by calculating a CHA_2_DS_2_-VASc and modified HAS-BLED score for each individual, respectively [20]. Definitions used for the construction of these scores are in Appendix 4. Prescription of an OAC within 6 months preceding care home entry was also recorded. Data on medication prescriptions were obtained from the WLGP. A history of smoking and diagnoses of cancer, dementia, dyslipidaemia, pulmonary diseases and peptic ulcer disease were also established on care home entry using Read and ICD-10 codes to extract relevant data from the WLGP and PEDW, respectively.

### Outcomes

Outcomes of interest were to determine the prevalence of AF and temporal trends by year of care home entry, and the incidence and risk of any stroke (ischaemic, haemorrhagic or stroke of unknown origin), TIA, cardiovascular hospitalisation, major bleeding, MI, cardiovascular and all-cause mortality in care home residents, stratified by a diagnosis of AF at care home entry. Incident events and their respective dates were obtained from the PEDW and WLGP. Date of death was determined from the Office for National Statistics Annual District Death Extract [21].

### Statistical analyses

To standardise the pre-care home entry data extraction for analyses of temporal trends in AF prevalence, the most recent record of AF from the WLGP or PEDW dataset within 10 years prior to care home entry was extracted for all participants with a minimum of 10 years data coverage within the WLGP prior to moving to a care home. Participants were recorded to have AF if they had at least one record of AF within the 10 year pre-care home entry window. This was to ensure consistency in the recording of AF diagnoses, specifically for participants recruited later on in the study period. For example, a participant entering a care home at the start of the study period (2003) could have a maximum of 3 years prior data, but a participant entering at the end of the study period (2018) could have a maximum of 18 years prior data. Consequently, a history of AF may be more likely to be captured for the participant entering in 2018. Direct standardisation was used to calculate age- and sex- standardised AF prevalence estimates by year of care home entry (2010–2018). Generalised linear models adjusted for age and sex were used to calculate the annual and absolute change in prevalence of AF over time. Analyses were also performed to provide prevalence estimates for AF recorded at any time point prior to care home entry for residents with a minimum of 10 years prior data (Appendix 5).

The incidence and incident rate of any stroke (ischaemic, haemorrhagic, stroke of unknown origin), TIA, cardiovascular hospitalisation, major bleeding, MI, cardiovascular and all-cause mortality after care home entry was reported for all participants by history of AF on care home entry. The Fine-Gray competing risk model was used to estimate the risk (sub-distribution hazard ratio [sHR]) of each outcome using mortality as a competing risk. All participants were followed up until they died, moved out of Wales or until the end of study (31 December 2018). Analyses were adjusted for covariates known to be associated with the outcomes of interest. These were pre-agreed amongst authors (LAR, SLH, DAL, PEP, AAkp, GYHL). The main analyses were adjusted for age, sex, WIMD, AF, frailty, smoking history, dementia, pulmonary disease, cancer diagnoses, peptic ulcer disease, prescription of OAC (with or without antiplatelet therapy) within 6 months prior to care home entry, CHA_2_DS_2_-VASc and HAS-BLED risk assessment scores. A sensitivity analysis was performed by running the same competing risk model but adjusting for the individual CHA_2_DS_2_-VASc and HAS-BLED risk assessment score components because there is some overlap (Appendix 4). When covariates were reported categorically and continuously, continuous covariates were used preferentially in the analyses. Similar covariates were grouped accordingly (Appendix 4) [18]. Multicollinearity was assessed using the Variance Inflation Factor (Appendix 5). If covariates had a Variance Inflation Factor > 10, authors (LAR, SLH, DAL, PEP) discussed this to determine suitability of inclusion in the model. A secondary analysis was also performed using standard Cox regression adjusting for the same covariates (Appendix 5). All analyses were completed using Stata v.15 (StataCorp, College Station, Texas 77,845, USA).

#### Research ethics and information governance

 

#### Patient and Public Involvement statement

The SAIL Databank consumer panel includes member of the public and provided feedback that the study outcomes were of interest.

**Figure 1:**
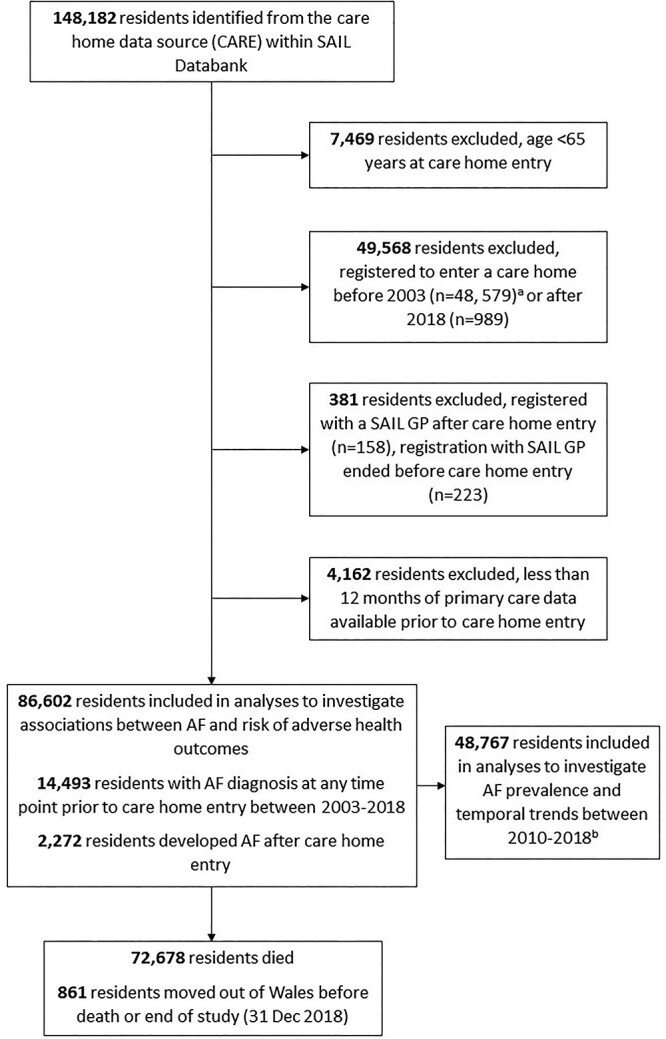
Flow diagram of study population selection from the SAIL Databank. AF, atrial fibrillation; GP, general practitioner; SAIL, Secure Anonymised Information Linkage. ^a^There were 34,045 residents who entered before the year 2000, complete linked data only available in SAIL from 2000 onwards. ^b^Analyses restricted to residents who had at least 10 years of primary care data captured within the SAIL Databank prior to standardise the pre-care home entry data extraction.

## Results

### Characteristics of study cohort on care home entry

Between 2003 and 2018, 86,602 people aged ≥65 years who had at least 12 months of primary care data captured within the SAIL Databank became new residents in care homes in Wales (Figure 1). The median (interquartile range [IQR]) age (years) of the cohort was 86.0 (80.8–90.6), and 27,661 (31.9%) were males. Of these, 14,493 (16.7%) were diagnosed with AF prior to care home entry, and 7,057 (48.7%) were prescribed OAC within 6 months prior to care entry. Residents with AF were slightly older (median age [IQR] 87.0 [82.6–91.2] vs. 85.7 [80.4–90.4]) and a higher proportion were male (35.2 vs. 31.3%). There was a higher prevalence of cardiovascular and cardiovascular-related comorbidities including stroke, TIA, MI, hypertension, heart failure, dyslipidaemia, peripheral vascular disease, venous thromboembolism, diabetes and renal disease in residents with AF. Demographic characteristics, frailty, stroke and bleed risk and comorbidities are reported in Table 1.

**Table 1:** Characteristics of adults aged ≥65 years within the SAIL Databank on care home entry (2003–2018), by history of AF prior to care home entry

Characteristics	All participants, *n* (%) (*n* = 86,602)	Participants without AF, *n* (%) (*n* = 72,109)	Participants with AF, *n* (%) (*n* = 14,493)
**Demographics**			
Age, median (IQR)	86.0 (80.8, 90.6)	85.7 (80.4, 90.4)	87.0 (82.6, 91.2)
Age category			
65–74 years	8,345 (9.6)	7,575 (10.5)	770 (5.3)
75–84 years	30,273 (35.0)	25,591 (35.5)	4,682 (32.3)
85–94 years	41,440 (47.9)	33,581 (46.6)	7,859 (54.2)
≥95 years	6,544 (7.6)	5,362 (7.4)	1,182 (8.2)
Male	27,661 (31.9)	22,558 (31.3)	5,103 (35.2)
WIMD quintile			
1	14,695 (17.1)	12,236 (17.1)	2,459 (17.1)
2	18,375 (21.4)	15,322 (21.4)	3,053 (21.3)
3	20,340 (23.7)	16,905 (23.6)	3,435 (23.9)
4	17,395 (20.2)	14,553 (20.3)	2,842 (19.8)
5	15,123 (17.6)	12,569 (17.6)	2,554 (17.8)
**Frailty**			
No frailty	28,870 (33.3)	27,006 (37.5)	1,864 (12.9)
Mild	26,505 (30.6)	22,791 (31.6)	3,714 (25.6)
Moderate	21,433 (24.7)	16,288 (22.6)	5,145 (35.5)
Severe	9,794 (11.3)	6,024 (8.4)	3,770 (26.0)
**Stroke risk**			
CHA_2_DS_2_-VASc score, median (IQR)	3 (3, 4)	3 (3, 4)	4 (3, 5)
**Bleed risk**			
HAS-BLED score, median (IQR)	2 (1, 3)	2 (1, 3)	3 (2, 3)
**Social history**			
Smoking history	20,775 (24.0)	16,779 (23.3)	3,996 (27.6)
Alcoholism	5,721 (6.6)	4,498 (6.2)	1,223 (8.4)
Heavy drinker	1,173 (1.4)	949 (1.3)	224 (1.5)
**Co-morbidities**			
Any stroke	11,375 (13.3)	8,466 (11.7)	2,929 (20.2)
Stroke (unknown)	2,811 (3.2)	2,193 (3.0)	618 (4.3)
Ischaemic stroke	7,832 (9.0)	5,600 (7.8)	2,232 (15.4)
Haemorrhagic stroke	1,669 (1.9)	1,333 (1.8)	336 (2.3)
TIA	3,095 (3.6)	2,299 (3.2)	796 (5.5)
Myocardial infarction	4,469 (5.2)	3,338 (4.6)	1,131 (7.8)
Heart failure	9,502 (11.0)	5,298 (7.3)	4,204 (29.0)
Alzheimer’s disease	1,711 (2.0)	1,549 (2.1)	162 (1.1)
Vascular dementia	3,345 (3.9)	2,793 (3.9)	552 (3.8)
Young onset dementia	<5 (<1)	<5 (<1)	<5 (<1)
Other dementia^a^	4,247 (4.9)	3,705 (5.1)	542 (3.7)
Asthma	5,687 (6.6)	4,473 (6.2)	1,214 (8.4)
COPD	8,647 (10.0)	6,853 (9.5)	1,794 (12.4)
Other pulmonary disease	41 (<1)	32 (<1)	9 (0.1)
Peptic ulcer	1,955 (2.3)	1,533 (2.1)	422 (2.9)
Diabetes	3,631 (4.2)	2,903 (4.0)	728 (5.0)
Renal disease	4,158 (4.8)	3,106 (4.3)	1,052 (7.3)
Liver disease	258 (0.3)	213 (0.3)	45 (0.3)
Cancer	12,615 (14.6)	10,379 (14.4)	2,236 (15.4)
Hypertension	31,850 (36.8)	24,883 (34.5)	6,967 (48.1)
Dyslipidaemia	8,202 (9.5)	6,437 (8.9)	1,765 (12.2)
Vascular disease	3,308 (3.8)	2,453 (3.4)	855 (5.9)
Aortic plaque	40 (<1)	32 (<1)	8 (0.1)
Major bleeding	10,942 (12.6)	8,307 (11.5)	2,635 (18.2)
Thromboembolism	1,886 (2.2)	1,422 (2.0)	464 (3.2)

AF, atrial fibrillation; CHA_2_DS_2_-VASc, stroke risk assessment scoring 1 point each for female sex, age 65–74 years, history of heart failure, diabetes, hypertension, vascular disease and 2 points each for history of stroke/transient ischaemic attack/venous thromboembolism and age ≥ 75 years; COPD, chronic obstructive pulmonary disease; HAS-BLED, bleeding risk assessment scoring 1 point each for age > 65 year, uncontrolled hypertension, liver disease, renal disease, harmful alcohol use, stroke history, prior major bleeding or a predisposition to bleeding, labile international normalised ratio and medication usage predisposing to bleeding; IQR, interquartile range; TIA, transient ischaemic attack; WIMD, Welsh Index of Multiple Deprivation

^a^Other or unspecified dementia

### Prevalence of AF at care home entry

**Figure 2:**
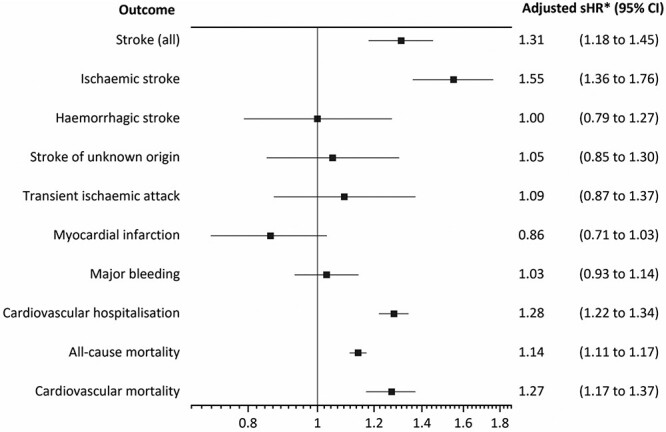
Risk of adverse health outcomes in care home residents aged ≥65 years with AF on care home entry included within the SAIL Databank (2003–2018), after adjustment of covariates, stroke and bleeding risk assessment scores—main analysis. CI, confidence interval; sHR, sub-distribution hazard ratio ^*^Adjusted for age, sex, WIMD, AF, frailty, smoking, dementia, pulmonary disease, cancer, peptic ulcer disease, prescription of oral anticoagulation (with or without concomitant antiplatelet therapy) within 6 months prior to care home entry, CHA_2_DS_2_VASc and HAS-BLED risk assessment scores, using mortality as a competing risk. Hazard ratios for all-cause and cardiovascular mortality not sub-distributed, standard Cox regression analysis.

There were 48,767 people aged ≥65 years who had at least 10 years of primary care data captured within the SAIL Databank prior to care home entry between 2010 and 2018, and 8,506 (17.4% [95% confidence interval [CI] 17.1–17.8]) had AF recorded 10 years before care entry. Prevalence [95% CI] of AF was higher in males than females (19.2% [18.6–19.8], *n* = 3,155 vs. 16.6% [16.2–17.0], *n* = 5,351), and increased with advancing age (65–74 years: 9.8% [9.0–10.7], 75–84 years: 16.3% [15.8–16.9], 85–94 years: 19.5% [19.0–20.0]) but stabilised in people ≥95 years (18.2% [17.1–19.5]). There was a non-significant increase in age- and sex-standardised prevalence (95% CI) of AF recorded within 10 years prior to care home entry from 16.79% (15.9–17.9) in 2010 to 17.0% (16.1–18.0) in 2018 (absolute change 2010–2018: 0.061, 95% CI −1.380 to 1.501, *P* = 0.93, annual change: 0.005, 95% CI −0.126 to 0.137, *P* = 0.94; see Table 2 and Appendix 5, Figure 1). Temporal trends in prevalence of AF recorded at any time point prior to care home entry were also reported (Appendix 5, Tables 1 and 2, Figure 2). Similarly, prevalence was found to be higher in males and increased with advancing age until people were 95 years or older. Without application of a time restriction for AF recording, overall age- and sex- standardised prevalence was higher (19.2%, 95% CI 18.8–19.5), and there was a significant increase in prevalence from 2010 to 2018 (absolute change 2010–2018: 2.53, 95% CI 1.04–4.02, *P* < 0.001, annual change: 0.33, 95% CI 0.19–0.47, *P* < 0.001).

**Table 2:** Prevalence estimates for AF recorded within 10 years prior to care home entry, limited to residents within the SAIL Databank aged ≥65 years with a minimum of 10 years data available prior to care home entry from 2010 to 2018

Year	Residents entering care home, *n*	Residents entering care home with AF, *n*	Crude prevalence (%) of AF (95% CI)	Age- and sex- standardised prevalence (%) of AF (95% CI)
2010	4,908	824	16.8 (15.8–17.9)	16.8 (15.9–17.9)
2011	4,960	871	17.6 (16.5–18.6)	17.68 (16.5–18.6)
2012	5,496	957	17.4 (16.4–18.4)	17.4 (16.4–18.4)
2013	5,567	990	17.8 (16.8–18.8)	17.7 (16.7–18.7)
2014	5,494	951	17.3 (16.3–18.3)	17.4 (16.4–18.4)
2015	5,768	1,025	17.8 (16.8–18.8)	17.7 (16.8–18.7)
2016	5,479	941	17.2 (16.2–18.2)	17.2 (16.2–18.2)
2017	5,344	966	18.1 (17.1–19.1)	17.9 (16.9–19.0)
2018	5,751	981	17.1 (16.1–18.1)	17.0 (16.1–18.098)
**Annual change** (95% CI), *P*-value	−	−	0.031 (−0.101 to 0.163), *P* = 0.65	0.005 (−0.126 to 0.137), *P* = 0.94
**Absolute change** (95% CI), *P*-value	−	−	0.269 (−1.176 to 1.714), *P* = 0.72	0.061 (−1.380 to 1.501), *P* = 0.93

AF, atrial fibrillation; CI, confidence interval

### Incidence and risk of adverse health events in care home residents with and without AF on care home entry

Care home residents were followed up for a median (IQR) of 538 days (166–1,167). Residents with AF on care home entry had significantly higher incident rates per 1,000 person-years of all adverse outcomes (any stroke, TIA, cardiovascular hospitalisation, major bleeding, MI, cardiovascular mortality and all-cause mortality, Table 3). The incident rates of any stroke and cardiovascular hospitalisation in residents with AF were almost double than those rates reported for residents without AF (any stroke: 25.42 [95% CI 23.51–27.48] vs. 14.97 [14.39–15.58], cardiovascular hospitalisation: 156.97 [151.76–162.37] vs. 83.77 [82.30–85.26]). There were 72,678 (83.9%) residents who died during the study period (2003–2018).

**Table 3:** Incidence and risk of stroke, TIA, cardiovascular hospitalisation, major bleeding and mortality in care home residents aged ≥65 years by AF status on care home entry (2003–2018)

	Number of events, *n* (%)	Incident Rate per 1,000 person-years (95% CI)	Unadjusted sub-distribution hazard ratio (95% CI), *P*-value	Adjusted sub-distribution hazard ratio^a^ (95% CI), P-value	Adjusted sub-distribution hazard ratio^b^ (95% CI), P-value
**Any stroke**					
No AF	2,437 (3.4)	14.97 (14.39–15.58)	1	1	1
AF	635 (4.4)	25.42 (23.51–27.48)	1.32 (1.21–1.44), *P* < 0.001	1.31 (1.18–1.45), *P* < 0.001	1.42 (1.29–1.57), *P* < 0.001
**Ischaemic stroke**					
No AF	1,391 (1.9)	8.51 (8.08–8.97)	1	1	1
AF	408 (2.8)	16.25 (14.75–17.91)	1.49 (1.33–1.66), *P* < 0.001	1.55 (1.36–1.76), *P* < 0.001	1.67 (1.48–1.89), *P* < 0.001
**Haemorrhagic stroke**					
No AF	503 (0.7)	3.05 (2.79–3.33)	1	1	1
AF	109 (0.8)	4.32 (3.58–5.21)	1.10 (0.89–1.35), *P* = 0.38	1.00 (0.79–1.27), *P* = 0.98	1.14 (0.91–1.42), *P* = 0.25
**Stroke of unknown origin**					
No AF	612 (0.8)	3.69 (3.41–4.00)	1	1	1
AF	137 (0.9)	5.34 (4.51–6.32)	1.12 (0.93–1.35), *P* = 0.24	1.05 (0.85–1.30), *P* = 0.67	1.13 (0.92–1.39), *P* = 0.23
**TIA**					
No AF	598 (0.8)	3.66 (3.38–3.97)	1	1	1
AF	120 (0.8)	4.79 (4.00–5.72)	1.01 (0.83–1.23), *p* = 0.90	1.09 (0.87–1.37), *P* 0.46	1.13 (0.91–1.40), *P* = 0.28
**Cardiovascular hospitalisation**					
No AF	12,359 (17.1)	83.77 (82.30–85.26)	1	1	1
AF	3,387 (23.4)	156.97 (151.76–162.37)	1.46 (1.41–1.52), *P* < 0.001	1.28 (1.22–1.34), *P* < 0.001	1.30 (1.24–1.35), *P* < 0.001
**Major bleeding**					
No AF	3,014 (4.2)	18.79 (18.13–19.48)	1	1	1
AF	640 (4.4)	26.03 (24.08–28.14)	1.07 (0.98–1.17), *P* = 0.12	1.03 (0.93–1.14), *P* = 0.58	1.07 (0.97–1.17), *P* = 0.17
**MI**					
No AF	1,087 (1.5)	6.63 (6.25–7.04)	1	1	1
AF	194 (1.3)	7.67 (6.66–8.83)	0.90 (0.77–1.05), *P* = 0.16	0.86 (0.71–1.03), *P* = 0.10	0.84 (0.71–1.00), *P* = 0.05
**All-cause mortality**					
No AF	60,356 (83.7)	364.57 (361.66–367.49)	1	1	1
AF	12,322 (85.0)	483.95 (475.45–492.59)	1.30 (1.27–1.33), *P* < 0.001^c^	1.14 (1.11–1.17), *P* < 0.001^c^	1.13 (1.10–1.52), *P* < 0.001^c^
**Cardiovascular mortality**					
No AF	4,815 (6.7)	29.00 (28.19–29.84)	1	1	1
AF	1,102 (7.6)	43.27 (40.78–45.91)	1.43 (1.34–1.53), *P* < 0.001^c^	1.27 (1.17–1.37) *P* < 0.001^c^	1.23 (1.14–1.32), *P* < 0.001^c^

AF, atrial fibrillation; CI, confidence interval

^a^Main analysis—sub-distribution hazard ratio adjusted for age, sex, WIMD, AF, eFI , smoking, dementia, pulmonary disease, cancer, peptic ulcer disease, prescription of oral anticoagulation (with or without antiplatelet therapy) within 6 months prior to care home entry, CHA_2_DS_2_VASc and HAS-BLED risk assessment scores

^b^Sensitivity analysis—sub-distribution hazard ratio adjusted for age, sex, WIMD, AF, eFI, smoking, dementia, pulmonary disease, cancer, peptic ulcer disease, prescription of oral anticoagulation (without antiplatelet therapy) within 6 months prior to care home entry and individual components that constitute CHA_2_DS_2_VASc and HAS-BLED risk assessment scores

^c^Hazard ratio not sub-distributed, standard Cox regression analysis

After adjusting for covariates and accounting for high mortality rates, when compared to residents without AF, those with AF had a significantly higher risk of having an incident stroke (adjusted sHR 1.31, 95% CI 1.18–1.45). When individual stroke subtypes were examined, the risk of haemorrhagic stroke or stroke of unknown origin was not significantly different between residents with or without AF, but there was a significantly greater risk of ischaemic stroke (adjusted sHR 1.55, 95% CI 1.36–1.76). Having AF on care home entry did not significantly increase the risk of TIA, major bleeding or MI, but those with AF were at a significantly higher risk of cardiovascular hospitalisation (adjusted sHR 1.28, 95% CI 1.22–1.34), cardiovascular mortality (adjusted HR 1.27, 95% CI 1.17–1.37) and all-cause mortality (adjusted HR 1.14, 95% CI 1.11–1.17) (Table 3 and Figure 2). The Variance Inflation Factor was <1.7 for all covariates (Appendix 5, Table 3). These findings were corroborated when individual components of the CHA_2_DS_2_-VASc and HAS-BLED risk assessments were adjusted for (Table 3 and Appendix 5, Figure 3). In this instance, the Variance Inflation Factor was <2.1 for all covariates (Appendix 5, Table 4).

In standard Cox regression analyses, the risk of any stroke, ischaemic stroke, cardiovascular hospitalisation, cardiovascular mortality and all-cause mortality were significantly higher in care home residents with AF. Residents with AF were also at a significantly higher risk of major bleeding (Appendix 5, Table 5 and Figure 4). When individual components of the CHA_2_DS_2_-VASc and HAS-BLED risk assessments were adjusted for, the risk of TIA and sustaining a stroke of unknown origin was also significantly higher in residents with AF (Appendix 5, Table 5 and Figure 5).

## Discussion

### Key findings

This is the largest study of care home residents aged ≥65 years that provides a comprehensive analysis of the prevalence of AF and adverse health outcomes associated with this condition. The principal findings from this study are as follows: (i) overall crude prevalence of AF in older care home residents was 17.4% (ii) over time, there was no significant change in crude or age- and sex-adjusted AF prevalence; (iii) AF prevalence was higher in men and increased with advancing age from 65 years onwards, stabilising in residents aged ≥95 years; and (iiii) AF was associated with a significantly higher risk of adverse health outcomes, including ischaemic stroke, cardiovascular hospitalisation, cardiovascular and all-cause mortality after adjusting for potential confounding factors.

### Comparison with literature

The Global Burden of Disease study in 2019 concluded that there has been a rise in the number of AF cases globally [22]. This was attributed to population growth and ageing, because the age-standardised prevalence of AF remained stable between 1990 and 2019 [22]. This study lends support to the generalisability of these findings exclusively to the care home population; age- and sex-standardised prevalence of AF remained stable between 2010 and 2018. This result should be cautiously interpreted. Advancements in AF screening technologies and increased population screening over time have improved the detection of occult AF. Therefore, it is possible that the stable prevalence of AF could suggest relative decreases of AF in the care home population that have negated the effect of increased case detection. Another interpretation of the finding is that diagnostic rates of AF in the care home setting may be lagging, highlighting a gap in care. From review of the literature, only one other care home study was identified that reported on prevalence of AF across different age groups and sexes [23]. The prevalence of AF was reported to be higher in men than women in 11,788 care home residents (14 vs. 11%, *P* = 0.012, respectively) and found to increase with advancing age in 2004 [23], thereby corroborating the findings of the present study.

Stroke and TIA are the major adverse health outcomes associated with AF [24–26]. AF has also been identified as an independent risk factor for MI and cardiovascular hospitalisation [27, 28]. To date, no study has verified these findings in an older care home population accounting for competing mortality. The median follow-up (time from care home entry to death, moving out of Wales or end of study) was 538 days for care home residents in this study. We are satisfied this provides us with an accurate estimation of incidence and risk of adverse health outcomes based on a report that concluded the length of stay in care homes (time from care home entry to death) in England is estimated to be 462 days [29]. The current findings demonstrate that older care homes residents with AF are more susceptible to adverse health outcomes including stroke and cardiovascular hospitalisation than residents without AF, even when higher mortality rates are accounted for. However, there was no statistically significant association between AF and TIA or MI in the older care home population.

The study’s findings reinforce the need for a holistic and integrated approach to optimise AF management in the care home population. The current gold-standard AF management strategy is the Atrial Fibrillation Better Care pathway [30]. However, it is not yet known if this is translatable to a real-world, prospective cohort of older people with AF in care homes. One pilot and feasibility study is currently underway to test this [31].

### Strengths and limitations

This study provides a pooled estimate of crude and age- and sex-adjusted AF prevalence that is inclusive of all types of care homes and residents by using a nationwide data linkage resource. Despite limiting our analyses to cases of AF recorded within 10 years prior to care home entry, we captured 91% of AF cases from 2010 to 2018. The main limitations pertain to the use of routinely collected data. First, international normalised ratio data were unavailable. Second, it is possible that some diagnoses (e.g. dementia) were missed using Read or ICD-10 codes by relying on positive recordings of diagnoses, or classified incorrectly. Third, it was not possible to provide a further breakdown of AF outcomes such as major bleeding based on the prescription of OAC or not because prescription data were not available for the entire study period, or based on falls because data were unavailable. Fourth, deprivation data were missing for a small proportion of residents (0.8%, *n* = 674) and demographic data on ethnicity were unavailable.

## Conclusion

This study provides an accurate estimation of AF prevalence, incidence and risk of adverse health outcomes exclusively in a population of older care home residents. It highlights the need for appropriate OAC prescription for stroke prevention and optimal management of cardiovascular co-morbidities, irrespective of frailty status and predicted life expectancy. Future research efforts should focus on improving the quality of routinely collected data in care home populations to help define ‘optimal’ AF management in older care home residents, and encompass AF classifications, prescription refill and falls data. Algorithms to identify care home populations from other routinely collected datasets would be valuable.

## Supplementary Material

aa-22-0771-File002_afac252
